# Development of gene expression-based risk score in cytogenetically normal acute myeloid leukemia patients

**DOI:** 10.18632/oncotarget.571

**Published:** 2012-08-18

**Authors:** Elias Bou Samra, Bernard Klein, Thérèse Commes, Jérôme Moreaux

**Affiliations:** ^1^ Groupe d’études des transcriptomes, Université MONTPELLIER 2; ^2^ INSERM, U1040, Montpellier, F-34197 France; ^3^ CHU Montpellier, Institute of Research in Biotherapy, Montpellier, FRANCE; ^4^ Université MONTPELLIER1, UFR Médecine, Montpellier, France

**Keywords:** gene expression-based risk score, cytogenetically normal-acute myeloid leukemia, prognostic gene signature, EVI1 expression

## Abstract

Patients with normal karyotype represent the single largest cytogenetic group of acute myeloid leukemia (AML), with highly heterogeneous clinical and molecular characteristics. In this study, we sought to determine new prognostic biomarkers in cytogenetically normal (CN)-AML patients. A gene expression (GE)-based risk score was built, summing up the prognostic value of 22 genes whose expression is associated with a bad prognosis in a training cohort of 163 patients. GE-based risk score allowed identifying a high-risk group of patients (53.4%) in two independent cohorts of CN-AML patients. GE-based risk score and EVI1 gene expression remained independent prognostic factors using multivariate Cox analyses. Combining GE-based risk score with EVI1 gene expression allowed the identification of three clinically different groups of patients in two independent cohorts of CN-AML patients. Thus, GE-based risk score is powerful to predict clinical outcome for CN-AML patients and may provide potential therapeutic advances.

## INTRODUCTION

Acute myeloid leukemia (AML) is a cytogenetically and molecularly heterogeneous disease characterized by accumulation of a variety of somatically acquired genetic aberrations in myeloid precursors, resulting in their clonal proliferation and maturation arrest. These genetic alterations are found in bone marrow or blood cells of approximately 55% of previously-untreated adults with AML and have long been recognized as independent predictors for clinical outcome, allowing the classification of patients into favorable, intermediate, and unfavorable prognostic groups [1]. However, no genetic aberrations have been identified in 45% of adult AML patients yet. These cytogenetically normal (CN) patients are usually assigned to intermediate prognostic group [2]. Over the past decades, several gene mutations such as internal tandem duplication (ITD) of the *FLT3* gene, mutations in the *NPM1* gene, partial tandem duplication of the *MLL* gene, mutations in the *CEBPA* gene, and changes in gene expression, such as overexpression of *BAALC*, *ERG*, *EVI1*, *MN1* and *CDKN1B,* have been discovered to strongly affect clinical outcome of CN-AML patients [3,4]. Twenty-four% of CN-AML patients show none of the aforementioned mutations, underlining the biological and clinical heterogeneity of this disease [5].

The development of high-throughput gene expression profiling (GEP) is of interest to improve risk classification of patients with CN-AML. Bullinger et *al.* [6]*,* by combining supervised and unsupervised data analysis from 40K cDNA microarrays, reported a 133-gene signature that split CN-AML patients into 2 groups with different survival. Radmacher et *al.* [7] confirmed the prognostic significance of this signature on an independent CN-AML cohort, using Affymetrix U133plus2.0 microarrays. Metzeler *et al*. [8] identified 66 genes, whose expression was prognostic for overall survival (OS), and defined a prognostic score based on this signature. Altogether, these studies emphasized the power of GEP data to predict outcome of CN-AML patients.

Based on our previous experience in building powerful risk scores in patients with malignant plasma cell disorders [9], we looked for whether this strategy could be applied to design gene expression (GE) based-risk score in CN-AML patients using publicly-available data. We report here the design of a GE-based risk score, involving 22 genes, whose value is strongly prognostic in 2 independent cohorts of CN-AML patients.

## RESULTS

### GE-based risk score in CN-AML

**Figure 1:**
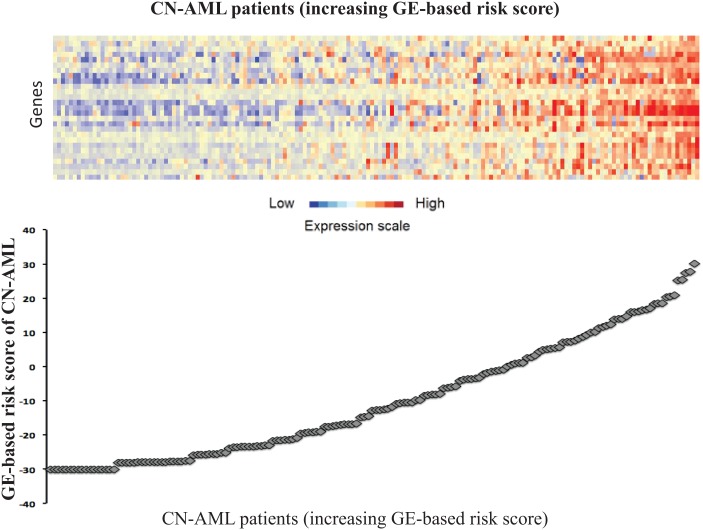
GE-based risk score in CN-AML Clustergram (upper part) of genes ordered from best to worst prognosis and samples ordered by increasing GE-based risk score (lower part) for CN-AML patients (N=163). The level of the probe set signal is displayed from low, deep blue to high, deep red gene expression.

**Figure 2:**
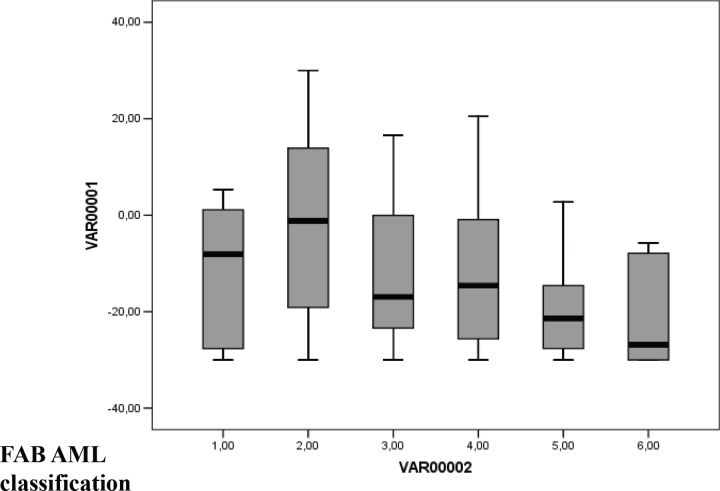
GE-based risk score in FAB CN-AML classification The GE-based risk score was investigated in the groups of the FAB classification AML in the CN-AML cohort of 163 patients (M0: Minimally differentiated acute myeloblastic leukemia; M1: Acute myeloblastic leukemia without maturation; M2: Acute myeloblastic leukemia with maturation; M4: Acute myelomonocytic leukemia; M5: Acute monocytic and monoblastic leukemia; M6: Acute erythroid leukemia). * Indicate that the score value is significantly higher in the group compared to all the patients of the cohort (*P* < .05). ** Indicate that the score value is significantly lower in the group compared to all the patients of the cohort (*P* < .05).

Using Maxstat R function and Benjamini-Hochberg multiple testing correction [10], 27 probe sets were found to be significantly associated with poor prognosis (adjusted *P* value <.05) (Table 1). These probe sets probed for 22 unique genes and 2 expressed sequence tag clones and were used to build the GE-based risk score. Figure 1 shows the variation of GE-based risk score along patients of the training cohort and the expression of the prognostic probe sets. With respect to AML FAB classification system, the GE-based risk score was significantly higher (*P* < 3.10^−3^) and lower (*P* < 1,8.10^−2^) in M1 and M5 subgroups, respectively (Figure 2).

**Table 1:** List of the 27 probe sets associated with poor prognosis in CN-AML patients Gene symbol, adjusted *P*-value and hazard ratios (HR) are given for each gene. Probe sets are sorted by decreasing HR.

Name	Gene Symbol	Adjusted *P* value	Hazard Ratio
217975_at	WBP5	0,0023	3,67
203860_at	PCCA	0,0057	3,67
227964_at	FRMD8	0,0407	3,46
237311_at	---	0,0009	3,37
203373_at	SOCS2	0,0011	3,33
201540_at	FHL1	0,0091	3,27
218086_at	NPDC1	0,0032	3,25
219922_s_at	LTBP3	0,0125	3,25
217820_s_at	ENAH	0,0101	3,22
215034_s_at	TM4SF1	0,0029	3,14
203372_s_at	SOCS2	0,0026	3,12
221973_at	LOC100506076 /// LOC100506123	0,0281	3,07
222803_at	PRTFDC1	0,0133	3,06
213056_at	FRMD4B	0,0065	3,00
212364_at	MYO1B	0,0426	2,96
204030_s_at	IQCJ-SCHIP1	0,0298	2,93
232752_at	LOC100287616	0,0130	2,91
209386_at	TM4SF1	0,0286	2,90
212387_at	TCF4	0,0106	2,88
243010_at	MSI2	0,0123	2,87
206950_at	SCN9A	0,0377	2,87
208798_x_at	GOLGA8A	0,0495	2,78
215071_s_at	HIST1H2AC	0,0248	2,73
227943_at	---	0,0355	2,73
206478_at	KIAA0125	0,0503	2,66
209387_s_at	TM4SF1	0,0425	2,66
212509_s_at	MXRA7	0,0444	2,65

**Figure 3:**
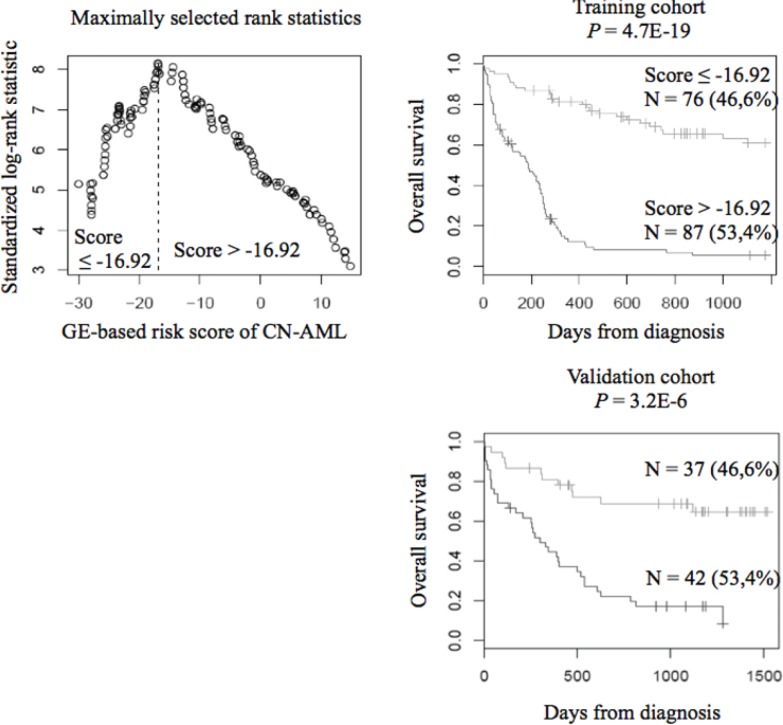
Prognostic value of GE-based risk score in CN-AML Patients of the training cohort (N=163) were ranked according to increased GE-based risk score and a maximum difference in OS was obtained with a score = -16.92 splitting patients in a high risk (53.4%) and low risk (46.6%) groups. The prognostic value of GE-based risk score was tested on an independent cohort of 79 patients (validation cohort). The parameters to compute the GE-based risk score of patients in the validation cohort and the proportions delineating the 2 prognostic groups were those defined with the training cohort.

When used as a continuous variable, GE-based risk score had prognostic value (*P* ≤ 10^−4^; data not shown). Patients of the training cohort (N=163) were ranked according to increased prognostic score, and for a given score value, the difference in survival of patients with a GE-based risk score ≤ score or > score was computed. A maximum difference in overall survival (OS) was obtained with a score = -16.92 splitting patients in a high-risk group of 53.4% of patients (prognostic score > −16.92) with a 6.2 months median OS and a low risk group of 46.6% of patients (prognostic score ≤ −16.92) with not reached median survival (Figure 3A). The prognostic value of our GE-based risk score was validated in an independent CN-AML patient's cohort (N=79) with a 9.9 months median OS in the high risk group and not reached median survival in the low risk group (Figure 3B).

Cox analysis was used to determine whether GE-based risk score provides additional prognostic information compared to previously-identified poor outcome-related markers such as *BAALC*, *ERG*, *MN1* or *EVI1* gene expression (supplementary Figure S1), and for gene signatures established by Bullinger's and Metzeler's groups [6,8]. Using univariate analyses, GE-based risk score, Bullinger's and Metzler's gene expression signatures, *BAALC*, *ERG*, *MN1* and *EVI1* gene expression were prognostic (*P* < .005; Table 2A). When compared two by two, GE-based risk score tested with *EVI1* expression remained significant (*P* < .0001; Table 2B). When all parameters were tested together, only GE-based risk score and *EVI1* gene expression kept prognostic value (Table 2C).

**Table 2:** Cox univariate and multivariate analysis of OS in CN-AML patients’ training cohort (N = 163) The prognostic factors were tested as single variable (A) or multi variables (B, C) using Cox-model. *P*-values and the hazard ratios (HR) are shown. NS, Not significant at a 5% threshold.

A.	Overall survival (n=163)	
Prognostic variable	HR	*P* value
GE-based risk score	6.79	<.0001
*BAALC* expression	1.99	.001
*ERG* expression	2.01	<.0001
*MN1* expression	2.49	<.0001
*EVI1* expression	2.06	.001
Metzeler's GEP score	3.41	<.0001
Bullinger's GEP signature	1.59	.01

B.	Overall survival (n=163)	
Prognostic variables compared two by two	HR	*P* value
GE-based risk score	6.51	<.0001
*BAALC* expression	1.12	NS
GE-based risk score	6.84	<.0001
*ERG* expression	.98	NS
GE-based risk score	6.05	<.0001
*MN1* expression	1.57	NS
GE-based risk score	7.57	<.0001
*EVI1* expression	2.37	<.0001
GE-based risk score	8.71	<.0001
Metzeler's GEP score	.73	NS
GE-based risk score	7.74	<.0001
Bullinger's GEP signature	.75	NS

C.	Overall survival (n=163)	
All prognostic variables	HR	*P* value
GE-based risk score	8.5	<.0001
*BAALC* expression	1.1	NS
*ERG* expression	1.0	NS
*MN1* expression	1.42	NS
*EVI1* expression	2.25	<.0001
Metzeler's GEP score	.52	NS
Bullinger's GEP signature	.59	NS

### Association of GE-based risk score and EVI1 expression as prognostic factor in CN-AML patients

**Figure 4:**
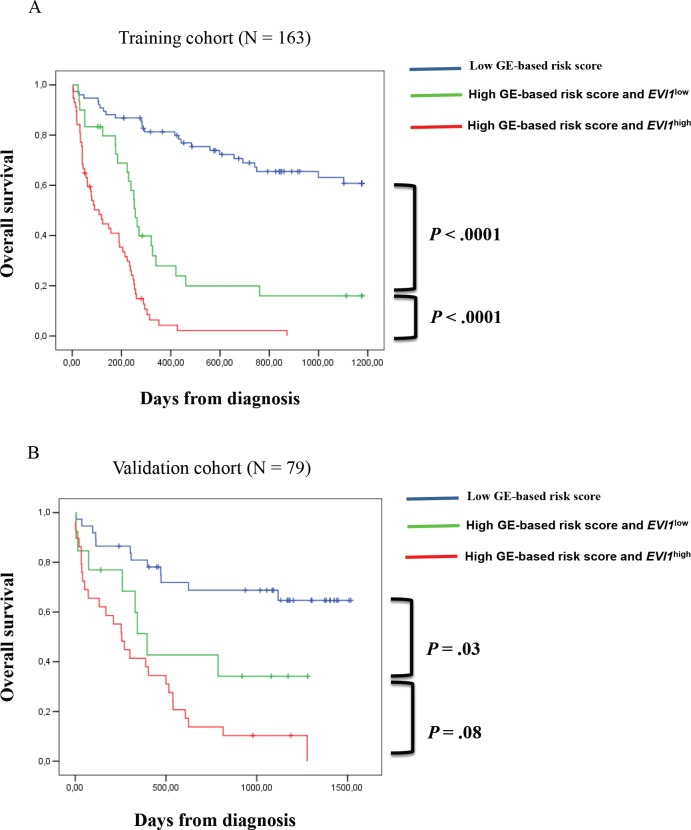
Association of GE-based risk score and EVI1 expression in CN-AML patients (A) Distribution of the patients and Kaplan-Meier estimates of overall survival in the training cohort of 163 patients of low risk score and *EVI1^low^* expression patients (blue), low risk score and *EVI1^high^* expression patients (black), high risk score and *EVI1^low^* expression patients (green) and high risk score and *EVI1^high^* expression patients (red). (B) Kaplan-Meier estimates of overall survival in the training cohort and validation cohort (C) of low risk score patients (blue), high risk score and *EVI1^low^* expression patients (green) and high risk score and *EVI1^high^* expression patients (red).

Since *EVI1* and GE-based risk score had independent prognostic information, they were combined to split patients into 3 groups with different OS. The first group comprised 40% of patients with low risk score, the second group 25% of patients with high risk score and *EVI1^low^* expression and the third group 35% of patients with high risk score and *EVI1^high^* expression. Patients of group 3 had the worst survival with 3.6 month median OS, patients of group 2 with high risk score and *EVI1^low^* expression had a median OS of 8.4 months and patients of group 1 had not reached median OS (Figure 4A). In the validation cohort of 79 CN-AML patients, median OS was not reached for group 1, was 13 months for group 2 and 8 months for group 3 (Figure 4B).

## DISCUSSION

Given the genetic heterogeneity of hematological malignancies, GEP studies have enabled the detection of new biologically and prognostically relevant subtypes of patients [9,11,12]. In the present study, we designed a GE-based risk score incorporating the prognostic information of 22 genes associated with poor OS in CN-AML patients. This risk score allowed splitting CN-AML patients of 2 independent cohorts into 2 groups: a high risk group with 6.2 or 9.9 months median OS and a low risk group with not reached median OS (Figure 3) [6,8].

Comparing the current list of 22 distinct genes with previously-published prognostic gene signatures, 2 and 17 of our identified target genes overlapped with the 133 and 86 survival predictors described by Bullinger *et al.* and Metzeler *et al.,* respectively [6,8] (supplemental Tables S1 A&B).

Besides the powerful prognostic value of this GE-based risk score, our current study highlights some pathways that could be involved in poor prognostic CN-AML. Among the 22 genes, the transcription factor *TCF4 (*T-cell factor 4) was shown to be a part of a gene set overexpressed in leukemic cells of acute T-cell leukemia/lymphoma patients [13] and to be associated with chemotherapy cross-resistance and treatment outcome in childhood acute lymphoblastic leukemia [14]. TCF4 protein is also known to interact with beta-catenin whose up-regulation has been observed in AML samples in association with poor prognosis [15]. Interaction of beta-catenin with TCF4 is critical in the activation of the cell cycle genes in response to upstream signals of Wnt/beta-catenin pathway. Interestingly, Tian *et al.* identified a new small molecule inhibitor named BC21 which inhibits TCF4/beta-catenin binding in colon cancer cells. BC21 blocks the clonogenic activity of colon cancer cells, down-regulates c-Myc and cyclin-D1 expression, and represents a new potential anticancer agent that targets TCF4/beta-catenin interaction [16]. This inhibitor could be of clinical interest in the high-risk group of CN-AML patients identified with our GE-based risk score. Overexpression of others genes included in our signature, *MSI2* (Musashi 2) and *SOCS2* (Suppressor of cytokines signaling 2), predicted unfavorable outcome in AML and chronic myeloid leukemia (CML) [17,18]. The two genes were also shown to be up-regulated in leukemia in the report from the Microarray Innovation in Leukemia (MILE) study group [19]. *MSI2* plays an important role in hematopoietic stem cells (HSC) proliferation and differentiation [20]. Enforced expression of *MSI2* in mice created a pre-leukemic phase [21] and its overexpression was found during transition from chronic to acute phase in a CML murine model. These findings were validated in CML patients [22]. Moreover, it has been demonstrated that MSI2 activates Notch signaling pathway, inhibiting translation of *Numb* mRNA, a negative regulator of Notch [20]. As well, among our prognostic predictors, we identified *TM4SF1* (Transmembrane 4 L six family member 1) and *SCN9* (Sodium channel, voltage-gated, type IX, alpha subunit). These markers were described as novel key regulators of tumor growth, invasion and metastasis in prostate cancer and were found to be markedly up-regulated in patients’ prostatic cells [23,24]. TM4SF1 is a tetraspanin-like membrane protein reported as a negative regulator of apoptosis in pleural mesothelioma tumor cells [25] and as a key regulator of endothelial cells function and angiogenesis that could represent an attractive therapeutic target [26].

Interestingly, when compared using multivariate analysis, only the current GE-based risk score and *EVI1* expression kept prognostic value. *EVI1* gene encodes a transcription factor with important role in normal hematopoiesis and leukemogenesis [27]. *EVI1* up-regulates cell proliferation through the activation of AP1 and by repression of transforming growth factor beta (TGF-beta) [28]. Moreover, high *EVI1* blocks differentiation through its interaction with transcription factors essential in hematopoiesis such GATA1 [29], SPI1 [30] and RUNX1[31]. The prognostic impact of *EVI1* expression has been a subject of debate since many years. Langabeer *et al.* [32] have demonstrated that *EVI1* deregulation is a relatively frequent event in AML, with no predictive impact on patients’ outcome. On the contrary, Lugthart et al. [33] showed that high *EVI1* levels predict adverse outcome among intermediate cytogenetic risk AML. In our study, this association allowed prognostic stratification of the high-risk group of patients who were either *EVI1^low^* or *EVI1^high^*. Furthermore, the prognostic impact of our GE-based score should be tested in the context of molecular mutations such as *FLT3* ITD and *NPM1* mutations [3].

Given the heterogeneity of CN-AML patients, the current GE-based risk score associated with *EVI1* expression would be of clinical value to identify patients who may benefit from intensive therapeutic strategies and to develop new targeted treatments in high risk patients.

## MATERIALS AND METHODS

### Patients

Gene expression microarray data from two independent cohorts of patients with CN-AML were used, the first cohort comprising 163 adult patients and the second one 79 adult patients. Pretreatment clinical characteristics of patients are shown elsewhere [8]. All patients received intensive chemotherapy. Affymetrix gene expression data are publicly available via the online Gene Expression Omnibus (http://www.ncbi.nlm.nih.gov/geo/)under accession number GSE12417. They were performed using Affymetrix HG-U133 A&B microarrays for first cohort of 163 patients and using Affymetrix HG-U133 plus 2.0 microarrays for the second cohort of 79 patients. Normalization of microarray data was performed using the variance stabilizing normalization algorithm, and probe set signals calculated by the median polish method. Quality control consisted of visual inspection of the array image for artifacts, assessment of RNA degradation plots, and inspection of rank-vs-residual plots after normalization and probe set summarization.

### Gene expression profiling and statistical analyses

Gene expression data were analyzed with SAM (Significance Analysis of Microarrays) [34], R [35] and Bioconductor [36] softwares. Hierarchical clustering was performed with the Cluster and Treeview softwares from Eisen [37].

### Selection of prognostic genes on the training set (cohort of 163 patients)

Probe sets were selected for prognostic significance using Maxstat R function and Benjamini Hochberg multiple testing correction [10] yielding 27 significant probe sets (Adjusted *P* value < .05; Table 1).

### Building gene expression (GE)-based risk score

To gather prognostic information of the 27 prognostic probe sets within one parameter, GE-based risk score of CN-AML was built as the sum of the beta coefficients weighted by ± 1 according to the patient signal above or below the probe set Maxstat value [10].

### Validation on the independent cohort of patients

The GE-based risk score of CN-AML was individually calculated and patients grouped according to the prognostic models and cut-offs from the training cohort. The prognostic value of this scoring was evaluated using log-rank statistics and Cox models.

### Statistical analyses

*BAALC*, *ERG*, *MN1* and *EVI1* gene expression was assessed using 222780_s_at, 211626_s_at, 205330_at and 221884_at Affymetrix probe sets, respectively. Their prognostic value was assessed using Maxstat R function (Supplementary Figure S1). Computations were done with R.2.10.1 (http://www.r-project.org/) and bioconductor version 2.5. Cox analyses were performed with the SPSS version 12.0 software (SPSS, Chicago, IL, USA).

## Supplementary Figures and Tables




