# Supplementary material for: Development of gene expression-based risk score in cytogenetically normal acute myeloid leukemia patients
Source: Oncotarget. 2012 Aug 18;3(8):824–32. doi: 10.18632/oncotarget.571 (PMC3478459; doi:10.18632/oncotarget.571)
Supplement: Supplementary file 1 [file oncotarget-08-824-s001.docx]

**Development of gene expression-based risk score in cytogenetically normal acute myeloid leukemia patients - Elias Bou Samra**

**
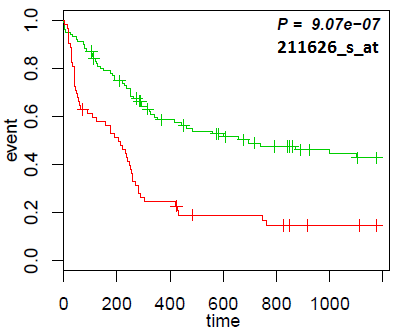

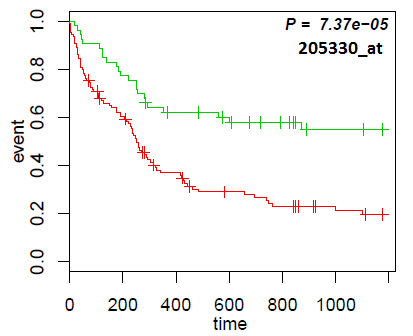

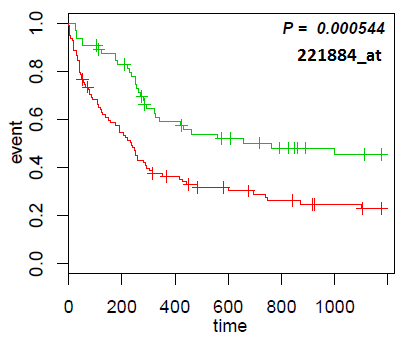

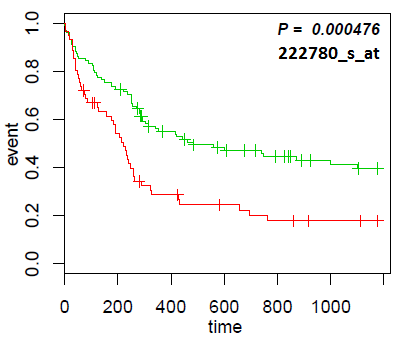
**

**Figure S1:** Overall survival in the training cohort of 163 CN-AML patients according to the poor outcome related markers. *BAALC*, *ERG*, *MN1* and *EVI1* gene expression was assessed using 222780_s_at, 211626_s_at, 205330_at and 221884_at Affymetrix probe sets, respectively. Red color represents high gene expression and green color represents low gene expression according to Maxstat R function analysis.

Overall survival

Overall survival

Overall survival

Overall survival

Days from diagnosis

Days from diagnosis

Days from diagnosis

Days from diagnosis

*MN1*

*EVI1*

63%

37%

*P* = .0004

*P* = 9E-7

62%

38%

*P* = 7E-5

*P* = .0005

67%

33%

60%

40%
