# Supplementary material for: Development of gene expression-based risk score in cytogenetically normal acute myeloid leukemia patients
Source: Oncotarget. 2012 Aug 18;3(8):824–32. doi: 10.18632/oncotarget.571 (PMC3478459; doi:10.18632/oncotarget.571)
Supplement: Supplementary file 2 [file oncotarget-08-824-s002.docx]

**Development of gene expression-based risk score in cytogenetically normal acute myeloid leukemia patients - Elias Bou Samra**


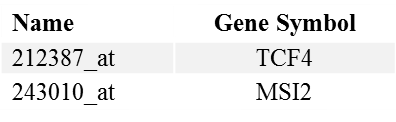

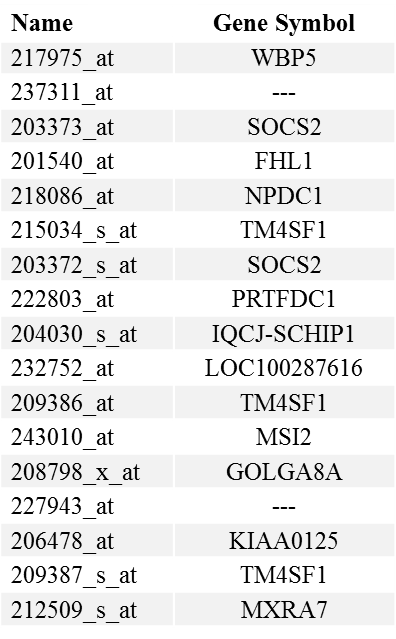


**B.**

**A.**

**Tables S1. A.** Common probe sets between our GE-based risk score and Metzeler’s risk score. **B.** Common probe sets between our GE-based risk score and Bullinger’s gene signature.
